# Genetic dissection of maize phenology using an intraspecific introgression library

**DOI:** 10.1186/1471-2229-11-4

**Published:** 2011-01-06

**Authors:** Silvio Salvi, Simona Corneti, Massimo Bellotti, Nicola Carraro, Maria C Sanguineti, Sara Castelletti, Roberto Tuberosa

**Affiliations:** 1 Department of Agroenvironmental Sciences and Technologies, University of Bologna, viale Fanin 44, 40127 Bologna, Italy; 2 Department of Horticulture and Landscape Architecture, 625 Agriculture Mall Drive, Purdue University, West Lafayette, IN 47907, USA

## Abstract

**Background:**

Collections of nearly isogenic lines where each line carries a delimited portion of a donor source genome into a common recipient genetic background are known as introgression libraries and have already shown to be instrumental for the dissection of quantitative traits. By means of marker-assisted backcrossing, we have produced an introgression library using the extremely early-flowering maize (*Zea mays *L.) variety Gaspé Flint and the elite line B73 as donor and recipient genotypes, respectively, and utilized this collection to investigate the genetic basis of flowering time and related traits of adaptive and agronomic importance in maize.

**Results:**

The collection includes 75 lines with an average Gaspé Flint introgression length of 43.1 cM. The collection was evaluated for flowering time, internode length, number of ears, number of nodes (phytomeres), number of nodes above the ear, number and proportion of nodes below the ear and plant height. Five QTLs for flowering time were mapped, all corresponding to major QTLs for number of nodes. Three additional QTLs for number of nodes were mapped. Besides flowering time, the QTLs for number of nodes drove phenotypic variation for plant height and number of nodes below and above the top ear, but not for internode length. A number of apparently Mendelian-inherited phenotypes were also observed.

**Conclusions:**

While the inheritance of flowering time was dominated by the well-known QTL *Vgt1*, a number of other important flowering time QTLs were identified and, thanks to the type of plant material here utilized, immediately isogenized and made available for fine mapping. At each flowering time QTL, early flowering correlated with fewer vegetative phytomeres, indicating the latter as a key developmental strategy to adapt the maize crop from the original tropical environment to the northern border of the temperate zone (southern Canada), where Gaspé Flint was originally cultivated. Because of the trait differences between the two parental genotypes, this collection will serve as a permanent source of nearly isogenic materials for multiple studies of QTL analysis and cloning.

## Background

The production and the phenotypic analysis of pairs of nearly isogenic lines (NILs) differing only for the allele constitution at given chromosome regions provides the opportunity to test for the presence at such regions of genetic factors involved in the inheritance of a quantitative trait [1,2]. In comparison with Quantitative Trait Locus (QTL) analysis carried out based on classical biparental mapping populations such as F_2_, recombinant inbred lines (RILs), etc., this should in principle enhance the statistical power of QTL detection by eliminating the blurring effect of multiple, and possibly interacting, segregating QTLs. A collection of NILs, each one differing from a reference recipient genotype for a known limited chromosome region, and altogether representing most of a donor genome, is known as introgression library (IL) [3,4]. In an IL, the donor genome is usually provided by an interfertile accession (usually a landrace or a wild relative), while a breeding elite strain is used as the recipient genetic stock. The process of IL production invariably involves some backcrossing scheme with the assistance of marker surveys during or after the backcross. ILs have been produced for a number of model and crop plant species (Reviewed in [5]; see also [6,7]), and even for model animal species such as mouse [8] and *Caenorhabditis *[9]. A pair of fully reciprocal IL populations were produced in *Arabidopsis *[10], with the two accessions used once as donor and once as recipient. One IL was described in maize involving two inbred lines, Tx303 and B73, as donor and recipient genotypes, respectively [11].

An IL enables moving and testing alleles from wild or landraces accessions into the elite gene pool of a crop, thus making possible their exploitation in plant breeding [4]. Accordingly, introgression lines belonging to partial or complete IL were proven to have breeding potential in cotton [12], maize [13], rice [14] and tomato [15]. Additionally, IL lines played a major role in enabling the positional cloning of major QTLs (eg. [16,17]), by providing the starting plant material where the genetic effect of the target QTL could be followed as any other Mendelian locus.

Here we describe the general features and the initial phenotyping of a maize intraspecific IL obtained using Gaspé Flint as the donor genotype and the elite line B73 as the recipient genotype. Gaspé Flint is a variety belonging to the Northern Flint maize race group [18], which was cultivated by American Native populations in southeastern Canada [19]. It is virtually the earliest known maize genotype and such earliness is the basis of its adaptation to the very short summer growing season of Canada. One of the genetic determinants of Gaspé Flint extreme earliness, the *Vegetative to generative transition1 *(*Vgt1*) QTL [20,21] has already been identified by positional cloning and shown to correspond to a non-coding, enhancer-like regulatory element of the AP-2 class transcription factor *ZmRap2*.7 [22]. The herein described B73 × Gaspé Flint IL lays the foundations for the genetic and molecular characterization of additional genetic determinants of flowering time and other traits of agronomic and adaptive importance in maize.

## Results

### Features and coverage of the introgression library

**Figure 1:**
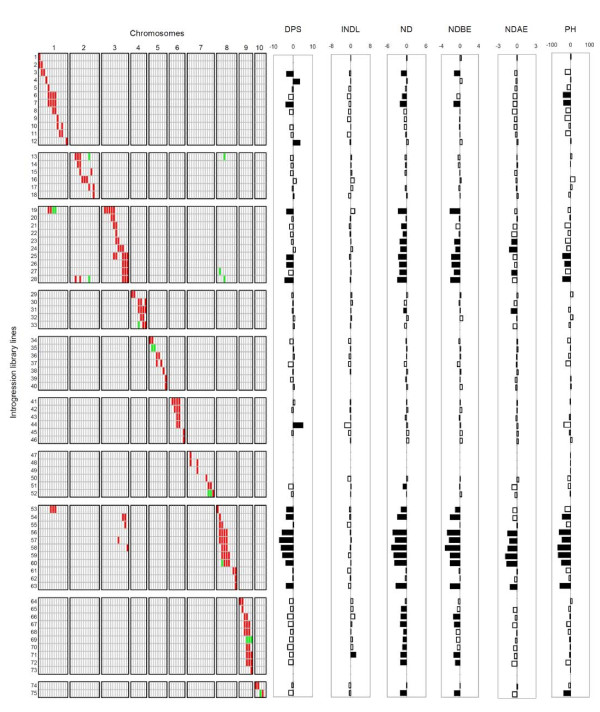
**Graphical genotype and QTL effect**. (A) Graphical genotype of the B73 × Gaspé Flint introgression library (IL). IL lines are represented horizontally and chromosome positions (polymorphic SSR markers as reported in Figure 2) are indicated vertically. Red and green rectangles indicate homozygous and heterozygous Gaspé Flint introgression, respectively. (B) Phenotypic differences between IL lines and B73, represented as horizontal columns. Black columns indicate IL lines significantly different from B73 (*P *< 0.05). Units are 'no. of days from planting' for days to pollen shed (DPS), 'cm' for internode length (INDL), 'node number' (ND), 'node number' below the top ear (NDBE), 'node number' above the top ear (NDAE) and 'cm' for plant height (PH).

The IL was produced following an SSR-based marker-assisted backcross procedure (Summarized in Methods) started from the cross B73 × Gaspé Flint. The genotypic composition of the 75 IL lines is shown in Figure 1A. Among these lines, 66 showed a single introgression, eight showed one additional introgression on a different chromosome and one showed two additional introgressions. The average introgression length, including lines with multiple introgressions, was 43.1 cM per lines (ca. 2.4% of the maize genome) and ranged between 4.5 and 104.0 cM (Table 1). Most of the lines carried homozygous introgressions, although partial heterozygosity was observed at six lines and total heterozygosity at two lines (ILL35 and ILL69). The majority of the lines carried unique introgressions, with the exception of six pairs of lines (ILL6 and ILL7, ILL43 and ILL44, ILL45 and ILL46, ILL62 and ILL63, ILL67 and ILL68, ILL71 and ILL72), where two lines per region were identified as showing similar introgressions. For each of these pair of lines, the second line was maintained in the IL set because derived from a partially different pedigree (i.e. from different BC_1 _- BC_4 _plants) within the IL backcross, implying that the two lines could carry different crossover events at the target introgression or different hidden introgressions. Additional redundancy of Gaspé Flint introgressions was intentionally maintained at bin 8.04-05 (covered by five different lines), bin 3.05-07 (four lines) and bin 9.03-04 (seven lines), which are sites of major flowering time QTLs (see below), in order to provide enhanced opportunities for further genetic investigations. Additional details about IL composition are reported in Table 1.

**Table 1:** Main features of the B73 × Gaspé Flint introgression library

IL lines characteristics		% of maize genome ^a^
IL lines (No.)	75	
Mean length of introgression in frame (cM)	38.5	2.1
Range of introgression length 'in frame' (cM)^b^	4.5 - 104.0	0.3 - 5.8
IL lines with completely homozygous introgression (No.)	68	
IL lines with partially homozygous introgression (No.)	6	
IL lines with completely heterozygous introgression (No.)	1	
IL lines with verified additional introgressions (No.)	9	
Mean length of verified additional introgressions (cM)	34.7	
Mean length of total introgression per line (cM)	43.1	2.4

^a ^Based on the 'Genetic 2008' maize reference map length of 1,805 cM http://www.maizegdb.org/map.php.

^b ' ^In frame' introgressions include one introgression fragment per line only. Additionally, redundant introgressions are excluded.

Among the 173 informative SSR markers, 101 showed a polymorphism between B73 and Gaspé Flint. Although monomorphic and polymorphic SSRs alternated along the chromosomes, regions with contiguous monomorphic SSRs were observed. Such regions hampered the recovery of the corresponding Gaspé Flint chromosome segments. By arbitrarily considering only segments with four or more contiguous monomorphic SSR, we identified six chromosome regions (Table 2), for a total of 220.5 cM, corresponding to 12.2% of the maize reference linkage map (see Methods). Such chromosome portions were technically non-representable within the library genome, at least with the markers used here. Given the low-resolution power of the standard agarose-gel electrophoresis utilized herein, the absence of polymorphism does not necessarily imply identity of nucleotide sequence between Gaspé Flint and B73 at such chromosome regions.

**Table 2:** Chromosome regions with four or more adjacent monomorphic SSR markers between B73 and Gaspé Flint

Chromosome bin	Markers included (No.) ^a^	Marker interval	cM
3.08-3.10	5	mmc0251-umc2048	66.5
4.02-4.04	8	umc1294-umc2206	30.9
5.00-5.01	6	umc1491-umc1781	35.2
6.01-6.02	4	bnlg1371-phi077	34.2
8.07-8.08	4	umc2014-umc1384	22.0
9.02-9.03	6	umc2219-umc1191	31.7

		Total	220.5

		Total (% maize genome)	12.2

^a ^Full markers list is provided in Additional file 6.

The non-overlapping fraction of the Gaspè Flint genome represented in the library corresponded to 1207.1 cM or to 66.9% of the maize reference map, which rose to 76.2% if we only consider the genome portion found polymorphic based on SSR profiles. Table 3 summarizes the IL coverage by chromosome.

**Table 3:** Chromosome coverage of the B73 × Gaspé Flint introgression library

Chromosome	Length	Coverage		Polymorphic portion	Coverage of polymorphic portion
	(cM)	(cM)	(%)	(%)	(%)
1	286	236.9	82.8	100.0	82.8
2	183	91.7	50.1	100.0	50.1
3	211	137.0	64.9	68.5	94.8
4	189	130.4	69.0	83.7	82.4
5	173	131.5	76.0	79.7	95.4
6	145	67.6	46.6	76.4	61.0
7	158	92.0	58.2	100.0	58.2
8	160	114.9	71.8	86.3	83.3
9	164	112.6	68.6	80.7	85.1
10	136	92.7	68.2	100.0	68.2

Maize genome	1,805				

Whole IL		1,207.1	66.9		76.2

Homozygous introgressions		1,172.3			

Heterozygous introgressions		34.8			

With the only aim to support and verify the QTL analysis results based on the IL population, we additionally characterized two small populations, a BC_1 _(88 plants) and an F_2 _(65 plants) both derived from B73 × Gaspé Flint crosses (see Methods).

### Phenotypic analysis

Table 4 lists the phenotypic traits (and corresponding acronyms) analysed in this study. As expected, Gaspé Flint showed much lower ND, DPS and PH values (10.7 nodes, 45.0 days and 106 cm, respectively) when compared to B73 (20.2 nodes, 74.7 days and 223 cm) (Table 4). Additionally, Gaspè Flint showed proportionally lower values for NDAE and NDBE (3.1 and 7.6 nodes) when compared with B73 (6.1 and 14.1 nodes, respectively), and no significant difference was observed for PNDBE. On the other hand, Gaspé Flint showed significantly higher EARN and INDL (3.4 and 15.8 cm) than B73 (1.5 ears and 13.8 cm, respectively). The B73 × Gaspé Flint F_1 _hybrid showed intermediate values between the parental genotypes for all traits except PNDBE for which no significance difference was observed, and for INDL for which it was shown to be significantly (*P *< 0.01) higher than B73 and Gaspé Flint (23.9, 13.8 and 15.8, cm respectively).

**Table 4:** Summary of phenotypic values for B73, F1 B73 × Gaspé Flint and Gaspé Flint, and trait heritability (h2)

Trait	Acronym (unit)	B73	F_1_	Gaspé Flint	***h^2 ^***(%)^a^
Days from planting to pollen shed	DPS (days)	74.7	60.7	45.0	88.8
Number of ears	EARN (count)	1.5	1.9	3.4	61.5
Growing degree unit	GDU (unit)	646	458	313	89.1
Internode length	INDL (cm)	13.8	23.9	15.8	75.3
Number of nodes	ND (count)	20.2	12.1	10.7	98.2
Number of nodes above the top ear	NDAE (count)	6.1	4.4	3.1	88.0
Number of nodes below the top ear	NDBE (count)	14.1	7.7	7.6	95.9
Plant height	PH (cm)	223.3	193.8	106.0	92.1
Proportion of nodes below the top ear	PNDBE (rate)	0.7	0.6	0.7	57.0

^a ^Computed based on the introgression library field experiment.

The majority of the IL lines had DPS, GDU, ND, NDBE and PH values close to B73 and only mildly skewed distributions toward the Gaspé Flint values were observed, accordingly with the recovery of most of the B73 genome (and therefore QTL alleles) in all lines (Additional file 1). ND, NDAE and NDBE values were non-normally distributed (*P *< 0.01). EARN and ND resulted non-normally distributed in the BC_1_, similarly to EARN, ND and ND-related traits in the F_2_.

The BC_1 _population showed a DPS frequency distribution shifted to lower values when compared with the F_2 _population (Additional file 1). The shift was likely observed because the BC_1 _population was grown later in the summer, in conditions of higher mean temperatures (not shown). As a confirmation, the shift disappeared when GDU were considered instead of DPS (Additional file 1). The ANOVA (or Kruskal-Wallis test), evidenced significant variation among IL lines (*P *< 0.001; not shown) for all traits. Broad sense heritability values ranged between 0.57 for PNDBE to 0.98 for ND (Table 4). Generally, plant (for F_2 _and BC_1_) or line (for IL) values were within parental values and little or no transgressivity was observed for the three populations with the exceptions of PH and INDL (Additional file 1). For PH and INDL, transgression was observed in the F_2 _and BC_1 _populations, with values higher than the high parent. This type of transgression (beyond the high-value parent) for plant height and related phenotypes is not unexpected given the inherent heterozygosity of F_2 _and BC_1 _populations that typically positively influences hybrid vigor. The transgressivity observed in the IL population is specifically treated in the QTL section.

### Correlation among traits

Across the three populations, ND was strongly positively correlated with NDBE, NDAE, DPS and PH. Additionally, ND was negatively correlated with INDL in the F_2 _and BC_1 _populations, while this correlation was not significant within the IL (Table 5 and Additional files 2 and 3). GDU and DPS were highly correlated (*r *= 0.99). PH was correlated (positively) with all traits except EARN and PNDBE. The genotypic and phenotypic correlation matrices based on the IL experiment provided almost identical results (Table 5).

**Table 5:** Genotypic (above diagonal) and phenotypic (below diagonal) correlations between traits based on the B73 × Gaspé Flint introgression library

	EARN	DPS	GDU	INDL	ND	NDAE	NDBE	PH	PNDBE
EARN	-	0.10	0.09	0.01	0.10	-0.02	0.23*	0.14	0.44*
DPS	0.07	-	0.99**	-0.09	0.89**	0.78**	0.91**	0.71**	0.18
GDU	0.07	0.99**	-	-0.08	0.89**	0.78**	0.91**	0.71**	0.17
INDL	-0.06	-0.10	-0.09	-	-0.11	0.01	-0.09	0.40**	-0.18
ND	0.08	0.82**	0.83**	-0.13	-	0.92**	0.98**	0.86**	-0.11
NDAE	-0.10	0.65**	0.66**	-0.09	0.88**	-	0.84**	0.81**	-0.34*
NDBE	0.17	0.83**	0.83**	-0.14	0.97**	0.73**	-	0.83**	0.23*
PH	0.06	0.66**	0.66**	0.46**	0.82**	0.72**	0.79**	-	-0.02
PNDBE	0.35**	-0.03	-0.03	-0.04	-0.19	-0.64**	0.05	-0.17	-

*, **: significance at *P *0.05 and 0.01, respectively.

### QTLs for flowering time

**Figure 2:**
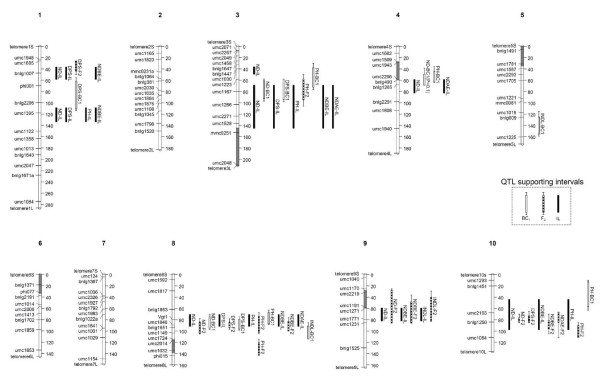
**Summary of QTLs identified in the B73 × Gaspé Flint Introgression Library (IL), BC_1 _and F_2 _populations**. QTLs are identified with the name of the traits (Table 1) and the population and represented as vertical bars (white, hatched or black, for BC_1, _F_2 _or IL, respectively) on the left of chromosomes. Large and thin bars indicate the 1-LOD and 2-LOD drop supporting intervals for the BC_1 _and the F_2 _QTL analyses, while for the IL QTLs the solid black bar indicates the region of Gaspé Flint introgression with significant phenotypic effect. Grey portions within chromosome bars indicate segments for which four or more SSR markers resulted monomorphic between B73 and Gaspé Flint. Chromosome representation includes polymorphic SSR markers utilized for IL production and the non-polymorphic SSRs delimiting the monomorphic regions between B73 and Gaspé Flint. Additionally, telomeres (based on the 'Genetic 2008' maize reference map available at http://www.maizegdb.org) are shown in order to provide indication of genome coverage.

Eight ND QTLs, on bins 1.02/3, 1.05/6, 3.03, 3.05/7, 4.04/5, 8.05, 9.03/4, and 10.04/5 were identified. In all cases, the direction of the genetic effect of ND and DPS QTLs was univocal, as previously noted for the well-characterized flowering time QTLs *Vgt1 *and *Vgt2 *[20-22], and in other studies [23]. This prompted us to name these flowering time loci as *qVgt*, followed by the bin number of their map location, with the exception of the QTLs on bins 8.04 and 8.05, for which we kept the former *Vgt1 *and *Vgt2 *acronyms. Summaries of QTL parameters and positions are provided in Table 6, Additional files 4 and 5, and Figure 2. A visualization of the phenotypic differences between each IL line and B73 is provided in Figure 1B.

**Table 6:** Features of the QTLs identified in the B73 × Gaspé Flint introgression library

Trait	QTL ^a^	Bin	cM ^b^	Marker interval ^c^	Effect ^d^	***P***
DPS		1.02-03	25.8 - 69.6	bnlg1007	-1.6	0.01
		1.05-06	108.7 - 134.0	umc1395	-1.6	0.001
		3.05-07	69.2 - 144.5	umc1167-umc1528	-1.5	0.001
		8.05	70.7-91.6	*vgt1*-umc1846	-2.4	0.001
		9.03-04	59.2 - 82.4	umc1271-umc1771	-1.1	0.001

GDU		1.02-03	25.8 - 69.6	bnlg1007	-21.1	0.01
		1.05-06	108.7 - 134.0	umc1395	-20.6	0.001
		3.05-07	69.2 - 144.5	umc1167-umc1528	-17.8	0.001
		8.05	70.7 - 91.6	*vgt1-*umc1846	-33.3	0.001
		9.03-04	59.2 - 82.4	umc1271-umc1771	-13.5	0.001

INDL		9.03-04	59.2 - 82.4	umc1271-umc1771	0.5	ns (0.10)

ND	*qVgt-1.02/3*	1.02-03	25.8 - 69.6	bnlg1007	-0.8	0.001
	*qVgt-1.05/6*	1.05-06	108.7 - 134.0	umc1395	-0.9	0.001
	*qVgt-3.03*	3.03	36.1 - 49.2	umc1030	-0.7	0.01
	*qVgt-3.05/7*	3.05-07	69.2 - 144.5	umc1167-umc1528	-1.1	0.001
	*qVgt-4.04/5*	4.04-05	58.8 - 82.6	bnlg490-bnlg1265	-0.4	0.05
	*Vgt1-Vgt2*	8.05	70.7 - 91.6	*vgt1*-umc1846	-2.0	0.001
	*qVgt-9.03/4*	9.03-04	59.2 - 82.4	umc1271-umc1771	-0.7	0.001
	*qVgt-10.04/5*	10.04-05	43.8 - 97.7	umc2163-bnlg1250	-1.0	0.001

NDAE		4.04-05	58.8 - 82.6	bnlg490-bnlg1265	-0.3	0.05
		3.05-07	69.2 - 144.5	umc1167-umc1528	-0.3	0.05
		8.05	70.7 - 91.6	*vgt1*-umc1846	-0.8	0.001

NDBE		1.02-03	25.8 - 69.6	bnlg1007	-0.6	0.01
		1.05-06	108.7 - 134.0	umc1395	-0.5	0.01
		3.05-07	69.2 - 144.5	umc1167-umc1528	-0.7	0.001
		8.05	70.7 - 91.6	*vgt1*-umc1846	-1.1	0.001
		9.03-04	59.2 - 82.4	umc1271-umc1771	-0.5	0.01
		10.04-05	43.8 - 97.7	umc2163-bnlg1250	-0.6	0.01

PH		1.05-06	108.7 - 134.0	umc1395	-17.4	0.01
		3.05-07	69.2 - 144.5	umc1167-umc1528	-14.0	0.05
		8.05	70.7 - 91.6	*vgt1*-umc1846	-26.0	0.001
		10.04-05	43.8 - 97.7	umc2163-bnlg1250	-17.4	0.01

^a ^QTL codes are given for the trait ND only (see text).

^b ^Position of the QTLs based on the Gaspé Flint introgressions position and length, as estimated on the reference maize map "Genetics 2008" http://www.maizegdb.org/map.php.

^c ^Markers or marker-intervals delimiting the Gaspé Flint introgressions at the QTL chromosome regions.

^d ^QTL genetic effects computed as (ILLs - B73)/2, where ILLs is the trait mean value of all IL lines sharing the same introgression at the QTL region.

The strongest QTL was identified at bin 8.04-8.05 in the IL, F_2 _and BC_1 _populations, accordingly with the known position of *Vgt1 *and *Vgt2 *[20]. The additive effect (a_i_, with i = IL or F_2_) attributed to *Vgt1-Vgt2 *complex locus in this study was estimated as a_IL _= -2.0 and a_F2 _= -1.5 nodes (sign indicates direction of effect induced by a Gaspé Flint allele). The Gaspé allele showed partial dominance. The *Vgt1-Vgt2 *region showed additional effects on DPS, NDBE, NDAE, and PH in the IL, on NDBE, DPS, and PH in the F_2_, and on DPS and PH in the BC_1_.

The second strongest effect QTL was *qVgt-3.05/7 *(a_IL _= -1.1). Additional significant effects were recorded for DPS, NDBE, NDAE, and PH. On the same chromosome, we identified *qVgt-3.03 *(a_IL _= -0.7). The BC_1 _QTL analysis confirmed the presence of flowering time QTL(s) on chr. 3, although the small population size likely precluded the clear separation of LOD peaks.

Two QTLs, *qVgt-1.02/3 *and *qVgt-1.05/6*, were identified on chr. 1. Such QTLs showed similar genetic effects for ND (a_IL _= ca. -0.8 and -0.9 nodes, respectively) and the same effect for DPS (a_IL _= ca. -1.6 days). The F_2 _LOD profiles indicated the presence of small effect QTLs for DPS at bin 1.02/3 and for PH at bin 1.05/6. The QTL analysis on BC_1 _identified a DPS QTL at an intermediate position between the two previous locations.

One QTL was identified on chr. 4 (*qVgt-4.04/5*) with a_IL _= -0.4 nodes. An ND QTL peak (although at sub-significance level, LOD peak = 2.6, threshold at LOD = 2.8) was identified at the same position in the BC_1 _population.

One QTL, *qVgt-9.03/4*, with a_IL _= -0.7 nodes was identified on chr. 9. The IL lines sharing similar chr. 9 introgressions showed a consistent although not significant effect on DPS as well. The presence of a QTL was confirmed by significant ND and NDBE LOD peaks and a consistent DPS LOD profile in the F_2 _population while the BC_1 _map did not properly cover this chromosome region.

On chr. 10, the *qVgt-10.04/5 *QTL was detected with a rather conspicuous additive genetic effect (a_IL _= -1.0 nodes) and with correlated effects on NDBE and PH and just below significance on DPS (not shown). Confirmation of map location was obtained with the F_2 _QTL analysis for ND, DPS, NDBE, NDAE and PH. No QTL was identified in this region in the BC_1 _population probably because of the reduced effect of the QTL in the BC_1_, since the B73 alleles showed partial dominance (at least for DPS, NDBE and NDAE). Alternatively, this QTL could be particularly sensitive to environmental cues, since the BC_1 _population was grown in a different year as compared to the IL and the F_2_, and with a late planting date. Interestingly, a QTL for photoperiod sensitivity was mapped right at bin 10.04 in several other studies [24-26].

The results of the QTL analysis for GDU were virtually equivalent to those for DPS (Table 6) and will not be discussed further.

### Other IL lines showing remarkable flowering phenotypes

A limited number of IL lines, namely ILL4, ILL12, ILL44, ILL51 and ILL63 (Figure 1B), showed flowering time-related phenotypes and yet their introgressed regions were not considered in the QTL summary because of lack of further experimental evidence from other lines or lack of coincidence with the F_2 _and the BC_1 _results. It should be noted that ILL4, ILL12 and ILL44, while not showing any ND change when compared to B73, flowered significantly later than the latter. Such subtle effect likely went undetected in the BC_1 _and F_2 _populations.

### QTLs for other traits

In keeping with the high phenotypic and genotypic correlation values, lines showing an effect on ND almost invariably influenced NDBE, with the only exception being the two minor QTLs *qVgt-3.03 *and *qVgt-4.04/5*. A similar trend was observed for NDAE, although in this case only three *Vgt *QTLs reached significance. The F_2 _QTL analysis confirmed the effects of *Vgt *QTLs on NDBE and NDBE, albeit at fewer QTLs, in accordance with the lower detection power of the F_2 _experiment.

The Gaspé Flint introgressions corresponding to *qVgt-1.05/6*, *qVgt-3.05/7*, *Vgt1-Vgt2 *and *qVgt-10.04/5 *significantly affected PH within the IL, with the Gaspé allele reducing PH. Additionally, PH mean values were similarly reduced, albeit not significantly, by the Gaspé allele at the other *qVgts*. The analyses of the F_2 _and BC_1 _identified two additional PH QTLs, at bins 8.06/8 and 10.01/4, respectively, which did not correspond to any *qVgts*. For the latter QTL, Gaspé Flint provided the allele with the positive effect.

One line (ILL71, bin 9.03/4) showed a significant effect on INDL, with the Gaspé Flint allele increasing the trait value (a_IL _= 1.0 cm; *P *< 0.05). Additionally, all other lines carrying introgressions at the same bin (corresponding to *qVgt-9.03/4*) showed a concordant direction of genetic effect (a_IL _= 0.5 cm; *P *< 0.10). The presence of the INDL QTL was confirmed in the F_2 _(a_F2 _= 2.1 cm). The lack of any detectable effect on PH at this chromosome region is probably caused by the balancing effect on PH due to a decrease of ND with a contemporary increase of INDL. Minor effect INDL QTLs were identified at bins 5.06/8 and 8.05/7 in the BC_1_, with Gaspé Flint providing the positive allele in both cases.

No EARN and PNDBE QTLs were detected in the three populations.

### IL lines with multiple *Vgt *introgressions

A number of IL lines were identified with trait values significantly different from B73 and carrying multiple introgressed *Vgt *QTLs. While their phenotypic values were not utilized to estimate the QTL effects, such lines are potentially useful for downstream analyses of QTL interaction or for marker-assisted applications. Examples of such lines are ILL25 (introgressions at *qVgt-3.03 *and *qVgt-3.05/7)*, ILL28, ILL54, ILL55 and ILL57 (introgressions at *qVgt-3.05/7 *and *Vgt1*).

### Qualitative traits

**Figure 3:**
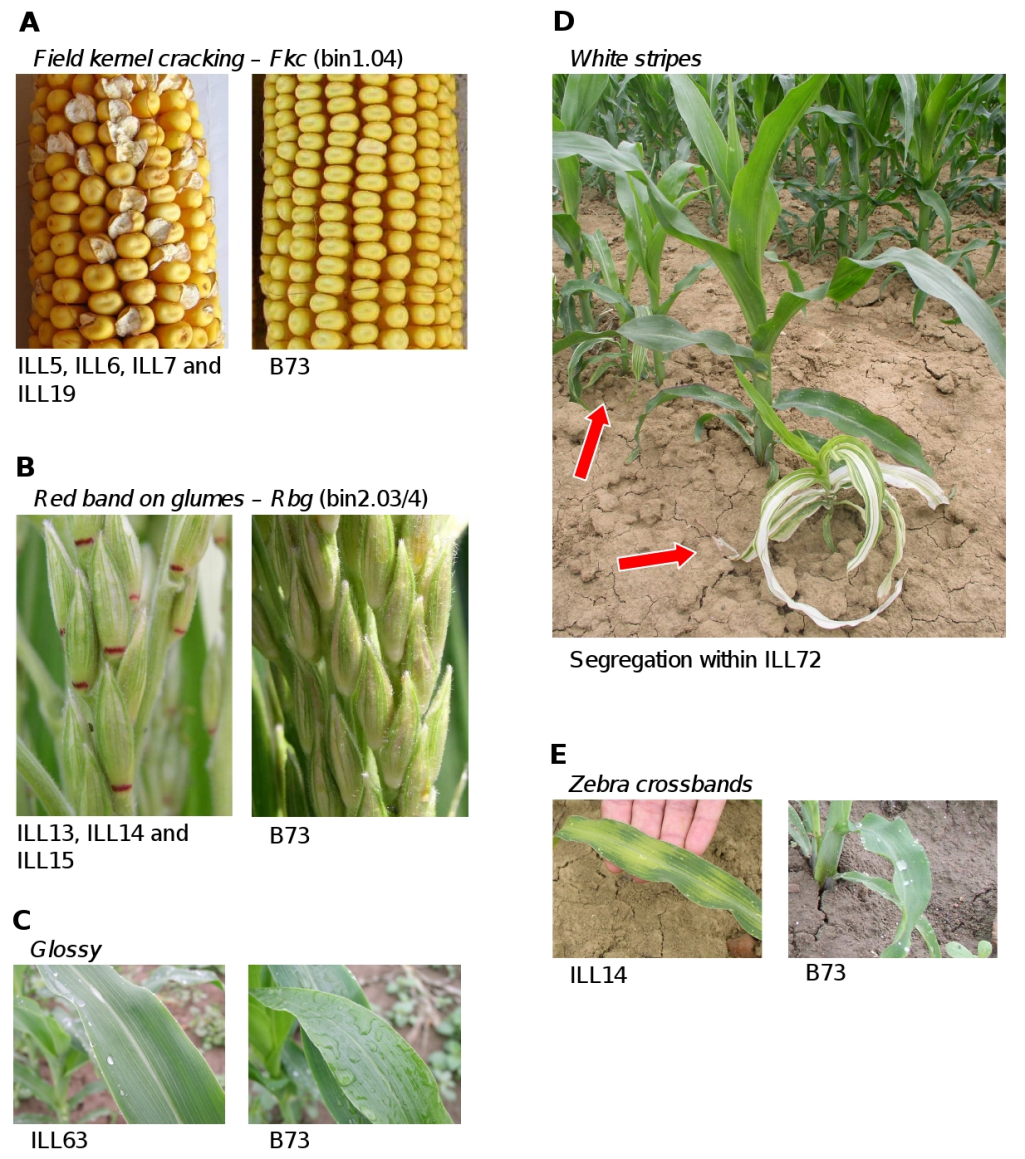
**Qualitative phenotypes observed within the B73 × Gaspé Flint introgression library**. (A) to (C) and (E): qualitative phenotypes as shown by relevant IL lines and comparison with B73 (right of each pair of images). (D): IL line plot showing phenotypic segregation with mutant/altered and wild-type plants.

A number of clearly qualitative phenotypes segregated within the IL. A locus (here tentatively named *Field kernel cracking, Fkc*) was found to influence the cracking (popping) of the kernel on the maturing ear (Figure 3A) and shown to map at bin 1.04 as confirmed by four IL lines carrying overlapping Gaspè Flint introgressions. A locus responsible for the presence of a pigmented red band on the tassel outer glumes (which we tentatively named *Red band on glumes*, *Rbg*) was confirmed by three IL lines carrying overlapping introgressions at bin 2.03/4 (Figure 3B). Other IL lines showed clear distinct phenotypes (*Glossy *for ILL63, *White stripes *for ILL72, *Zebra crossbands *for ILL14. Figure 3C-E). However, because each phenotype was observed based on one IL line only, the attribution of these loci to a given chromosome region remains uncertain. We further observed ILL4 as the only IL line with white cobs whereas all other lines and B73 had red cobs (not shown). This is in accordance with ILL4 introgression at bin 1.03 encompassing the *p1 *locus, known to provide pigmentation of the soft floral parts of the cob [27].

## Discussion

### Genetic basis of flowering time in maize

The molecular dissection of quantitative traits is moving quickly from QTL to trait mapping, which is not only mapping and cloning one or few QTLs but rather the identification of all the major components responsible for the genetic variability of a given trait in a crop species. Buckler and co-workers [28], using the nested-association mapping approach identified ca. 50 QTLs for flowering time at relatively high-resolution. However, even if such QTL map information can now be linked directly to the maize genome sequence [29] and thus to candidate genes, the identification of the causal genes or sequence features (also known as quantitative trait nucleotides - QTN [30]) remains unsolved, and will still require the development of targeted cross populations for positional cloning.

The IL lines and the QTL results described here provide a starting point for the positional cloning of seven additional flowering time QTLs, similarly to what has been achieved for *Vgt1*. Although the genetic effects estimated for these *Vgt *loci were considerably smaller than the *Vgt1 *one (ca. a = 1.8 ND [22]), the Mendelization of even the smallest effect QTL (*qVgt-4.04/5*, a = 0.4 ND), assuming a single causal gene per locus, could be obtained by phenotyping the segmental nearly-isogenic lines for ND based on a low level of replication (ca. three, on an eight-plant plot basis).

The full genetic dissection of the phenotypic differences between B73 and Gaspé Flint undoubtedly suffered by the incomplete coverage of the Gaspé Flint genome (ca 70%) and by the partial genotyping. The incomplete coverage could have prevented the identification of additional flowering time QTLs while the partial genotyping could have precluded the identification of multiple introgressions within IL lines. The latter situation could have lead to both false positive (the effect was wrongly attributed to an introgressed chromosome region while it was actually due to a QTL laying into a hidden introgression) and false negative (the effect of an identified introgression was counterbalanced by the effect of a hidden one), or more generally to biased estimation of effects. Such drawbacks were at least partially prevented by carrying out parallel QTL analyses on the B73 × Gaspé Flint-derived BC_1 _and F_2 _populations. In this regard, the comparison of the results of the QTL analyses from the three populations showed that all flowering time QTLs (in terms of ND and DPS) highlighted within the BC_1 _and the F_2 _populations were identified in the analysis of the IL. Such QTLs were the strongest in terms of genetic effect estimated in the IL, making unlikely that additional major QTLs went unnoticed within the IL.

Several important biological questions can be addressed based on the availability of this IL. As previously observed [20,23], we confirmed a high correlation between ND and DPS. Such correlation was also evident if direction and intensity of gene effect at the DPS and ND QTL are considered (Table 6). Correlation between two traits paralleled by the coincidence of QTLs and concurrent direction of QTL genetic effects have been recognized as indications of prevalent pleiotropy [31]. In our case, the causative QTNs at the flowering time QTLs would influence both ND and DPS. Because of the developmental architecture of cereals, this translates in allele variation influencing primarily ND and consequently DPS. However, linkage of different genes for ND and DPS at some of the flowering time QTLs cannot be excluded until molecular cloning of these QTLs will be accomplished. Additionally, we showed that QTLs for DPS were fewer than QTLs for ND and that QTLs for DPS coincided with QTLs for ND. Such observations imply that allelic variation at genes influencing the time of switch to the reproductive phase prevails over genetic variation for the rate of development (both plastochron and/or phyllochron, that is, the rate of phytomere differentiation and distension, respectively). Accordingly, genes known to be involved in maize plastochron (*corngrass1/mir156 *mapped on chr. 3, at 24.4 cM and *PLA3/Vp8*, mapped on chr. 1, at 243 cM [32,33]) map outside the confidence interval of the *Vgt *QTLs. The lack of any QTL involved in plastochron/phyllochron is puzzling. Perhaps strong allelic variation for plastochron/phyllochron genes is not compatible with extreme earliness and satisfactory crop production, in such a way that strong plastochron/phyllochron new early alleles were (are) eliminated unconsciously during the domestication and/or the breeding processes. Alternatively, in the early phases of maize domestication and expansion, variation at such genes (at least between B73 and Gaspé Flint) was lost. It is noteworthy that three lines (ILL4, ILL12, and ILL44) were identified that showed delayed DPS without significant effect on ND. Unfortunately, the existence of such QTLs was not corroborated by multiple IL lines and by the BC_1 _and F_2 _QTL results. For these lines, the Gaspé Flint allele contributed the late allele. Altogether, the existence of Gaspé Flint alleles delaying DPS without affecting ND cannot be completely excluded. The observed delay in flowering time could result from i) a general slow plant development or ii) specific delay of tassel and/or flower development and anther extrusion.

As expected and previously observed [23], ND variation also tightly drove variation for PH (the more numerous the phytomeres, the higher the plant) and NDBE (if apical dominance is constant or independently controlled from ND, a change in ND will directly reflect to NDBE). Unexpectedly, ND variation (and therefore *Vgt *QTLs) also correlated with NDAE, with the ND vs. NDAE phenotypic correlation *r *= 0.88 (*P *< 0.01) (Table 5). One explanation is that NDAE (therefore the extension of the apical dominance signal) is related to plant height by the presence of a root- or crown-originated acropetal promoting-signal counterbalancing the apical dominance basipetally driven by auxin [34]. Alternatively, co-segregation for QTLs influencing apical dominance along with flowering time is possible. As a matter of fact, the NDAE effect detected in this study at bin 3.05/7 coincides with a QTL previously reported for the same trait [23,35], and a well-supported candidate gene (*barren stalk1 *[36]). Additionally, the *Lfy *locus, influencing the number of leaves above the ear [37], maps on 3L [27].

Only bin 9.02/4 consistently showed an effect on INDL, based on IL and F_2 _QTL analyses. Interestingly, the gene *Dwarf3*, that codes for a P-450 cytochrome involved in gibberelline biosynthetic pathway [38] maps within such interval.

PH and INDL QTLs with positive Gaspé Flint allelic effect were identified in the BC_1 _and F_2 _populations and not in the IL (Additional files 4 and 5). Such result was likely the consequence of the residual heterozygosity of the BC_1 _and F_2 _populations, which drove the expression of heterotic effects on PH and INDL.

By producing and testing IL lines with introgressions at two or more loci, epistatic interactions among QTLs can be addressed in a (statistically) powerful way [39,40]. Within the B73 × Gaspé Flint genetic background and for flowering traits, the presence of such interactions can be anticipated. The ND genetic effects of all *Vgt *QTLs under a fully additive mode summed to 14.7 nodes where the B73 - Gaspé Flint difference was 9.5 nodes; on the contrary *Vgt *QTL effects on DPS summed to 18.2 days, whereas the B73 - Gaspé Flint difference was 29.7 days. Such discrepancy is likely the consequence of multiple epistatic effects between different *Vgt *QTLs, which could reflect upon ND and DPS in different ways. However, additional causes could be (i) the segregation between B73 and Gaspé Flint of additional flowering loci lacking the strict ND-DPS pleiotropism and not represented within the IL, and (ii) the effect on the Gaspé Flint phenotypic mean value of a dominance component originated by the inherent Gaspé Flint heterozygosity.

### Molecular bases of flowering time

This IL provides the opportunity to test the old hypothesis that the amount of nuclear DNA (C-value) influences flowering time. Maize genome size was shown to be negatively correlated with latitude and length of growing season [41,42] and selection for earliness was linked with a reduction of C-values [43]. Similarly, a correlation was found between the presence of knobs (cytologically-detectable centromere-related chromosome regions, known to contain a large number of repeat units [44]) and delayed flowering time [45]. Gaspé Flint has been repeatedly shown to have one of the lowest C-values among the genus *Zea *[46] and to carry the least number of knobs-resident DNA repetitive elements in comparison with other investigated inbreds [47].

A number of coincidences of *Vgt *QTLs with flowering time QTLs mapped in other studies were found. *Vgt1-Vgt2 *and *qVgt-10.04/5 *coincided with two of the three highly recurrent consensus QTLs identified in a recent survey of 441 flowering time QTLs [48] and four *Vgt *QTLs (*qVgt-1.05/6*, *Vgt1 *and *Vgt2*, *qVgt-9.03/4 *an *qVgt-10.04/5*) overlapped with 'hot-spot' QTLs identified after QTL meta-analysis [49]. Additionally, all the other *Vgt *QTLs mapped at regions of relatively high QTL density [47]. Part of the explanation for such coincidence is likely the redundant use of B73 and related inbred lines as parents in many experimental populations [48].

Beside *Vgt1*, no other *Vgt *locus has been resolved at the level of candidate sequence and/or QTN. However, evidence of genes possibly involved in flowering time and corresponding to *Vgt *loci (based on comparative and/or fine mapping) have been collected in at least four studies. An FT-like gene, *ZCN8*, co-mapping with *Vgt2 *(bin 8.04) was proposed as candidate based on the *FT *strict homology and pattern of expression profile [50]. *Glossy15*, an Ap2-like gene closely related with *ZmRap2*.7 and shown to regulate the juvenility traits and the time to flowering [51] co-maps with *qVgt-9.03*. *qVgt-1.05/6 *overlaps with *bif2*, a gene for which natural allelic variation has been correlated with flowering time and node number [52] and with an *SPL*-like gene implicated with flowering time in *Arabidopsis *[23]. The region of *qVgt-10.04/5 *harbors a photoperiod-sensitive locus shown to include a CTT-like gene homologous to a heading-date gene of rice [25].

### Genetics of adaptation to a short growing season environment

Our results showed that the extreme earliness of Gaspé Flint, if compared to standard Corn Belt lines, is due to allelic variation at several loci, generally detected in other crosses [48], one of which (*Vgt1*, in this study linked with *Vgt2*) with sizeable effect. As a next step, it would be valuable to verify to what extent Gaspé Flint earliness is due to the combination of early alleles commonly present in the maize germplasm or to unique early alleles. The first hypothesis seems to be supported by the results obtained from the positional cloning of *Vgt1*, the major of such flowering time QTLs. At *Vgt1*, Gaspé has been shown to carry a relatively common haplotype, shared even by medium-late Corn Belt dent lines [22], tropical germplasms and teosintes [53]. Additionally, the coincidence of the *Vgt *QTLs with many other flowering time QTLs supports the notion that Gaspé Flint alleles are present in other genetic backgrounds. Therefore, Gaspé Flint could be the prototype of an unconscious QTL-allele pyramidization process, carried out by pre-Columbian, Native American farmers to adapt a tropical species to cultivation at the northern limit of the temperate zone by recruiting alleles for earliness at a number of loci. The importance of the molecular verification of such hypothesis cannot be overemphasized, since QTL pyramiding is at the core of the current genomics-assisted breeding paradigm [5].

Nonetheless, it is possible that mutations contributing new early alleles arose uniquely in the gene-pool under selection for earliness, and such mutations remained confined in the Northern Flints. Recurrent mutation at the *sugary1 *locus has already been shown to have occurred during the process that led to the release of modern sweet maize inbreds and cultivars [54]. A high mutation rate was also hypothesized to be responsible for the linear response to selection for flowering time, in a selection experiment starting from a small, highly inbred maize population [55]. Additionally, the extreme plasticity of the maize genome caused by the activity of transposable elements at coding as well as at intergenic regions [56,57] makes it likely that changes at the regulatory region of flowering time genes could have caused early flowering, and could have recurrently been selected. Reinforcing this hypothesis, Gaspé Flint was already reported to show an unusually high rate of spontaneous mutations [58]. If confirmed, such high mutation rate could have provided the unique early alleles upon which selection for earliness acted.

The adaptation of maize, a tropical species, to the northern latitudes, and the molecular changes enabling it, can be set in a rather precise timeframe. Maize was domesticated from teosinte in the Balsas basin of southern Mexico ca. 6,000 - 10,000 years ago [59], then reached south western United States ca. 3,000 years ago and northern United States and Canada only 1,000 years ago, at the time when the very well-defined maize race group, the Northern Flint, originated [18,60]. The short early maize grown by the Penobscot Algonkins people in the Gaspé peninsula and described by the Canadian explorer Jacques Cartier in 1555 was likely Gaspé Flint [61]. The relatively late northward expansion of maize could have partially been caused by the complex genetic adaptations (e.g. extreme early flowering) required for the short growing season [18].

## Conclusions

The genetic basis of maize flowering time was investigated using an introgression library developed using the virtually earliest maize cultivar, Gaspé Flint, and the elite reference inbred line B73. At least eight chromosome regions, including the well-known flowering time locus *Vgt1*, showed allelic variation influencing flowering time. These detected flowering time QTLs are involved in determining the number of vegetative phytomeres developed by the maize plant before switching to reproductive phase and do not appear to influence the rate of development (i.e.: plastochron/phyllochron). Thus, reducing the number of phytomeres seems to have been the key developmental feature enabling the adaptation of a tropical crop species like maize to much northern environments such as southern Canada. Thanks to the genetic constitution of the plant material (nearly isogenic lines) here described, all QTLs have been isogenized and are thus available for fine mapping. We anticipate that the availability of this introgression library will further contribute to the dissection and identification of the molecular bases of the rather impressive adapting plasticity of maize.

## Methods

### Plant material and production of the library

Gaspé Flint is an open-pollinated cultivar belonging to the Northern Flint germplasm race group [18] and characterized by extreme earliness, low stature, high tillering and multiple ears. Seed of Gaspé Flint was kindly donated by Ronald Phillips, University of Minnesota, St. Paul, USA. B73 is an elite inbred line belonging to the Iowa Stiff Stalk Synthetic heterotic group [62]. From the starting cross (B73 × Gaspé Flint), two F_1 _plants were utilized in a backcross with B73. Following the production of 88 B73 × (B73 × Gaspé Flint) BC_1 _plants, each plant was sampled for DNA extraction and profiled with 140 SSR markers (see below). Based on this molecular profiling, a set of heterozygous target chromosome regions (approximately one per BC_1 _plant), marked by two SSR flanking markers, were defined. From such plants, four additional generations of backcross using B73 as recurrent genotype were carried out, followed by three cycles of selfing. For each backcross or self generation and for each backcross family, approximately 20 plants were grown in the field and genotyped for the two markers flanking the target chromosome region. From BC_2 _to BC_5_, for each family, one plant (the one with more seed) heterozygous at the target region was advanced to the subsequent backcross generation. At the BC_5 _stage, plants heterozygous for the target introgression were selfed, producing BC_5_F_2 _seed. Plants of each BC_5_F_2 _family were screened with the assigned flanking SSR in order to identify and self plants (one per family) carrying the homozygous Gaspé Flint introgression. BC_5_F_3 _families were then grown and selfed for seed increase and storage. The plants utilized for this experiment corresponded to BC_5_F_4 _whereas available IL seed stocks correspond to BC_5_F_4 _or BC_5_F_5_.

### Marker analysis

Genomic DNA was prepared from young leaf tissues as previously described [63]. A total of 180 SSR markers (Additional file 6) were utilized for searching polymorphisms between B73 and Gaspé Flint, 173 of which were informative and seven failed to do so. From the same set, SSR markers were selected for genotyping the IL. Primer information was downloaded from the Maize Genome Database at http://www.maizegdb.org. One additional marker, *Vgt1-Mite*, was utilized based on the information provided in [22]. PCR analyses for SSR were carried out as described [21] and separation and visualization followed standard agarose-gel electrophoresis protocols.

### Genome coverage and features of the IL

Position for all markers as well as the length of chromosomes and introgressions are given in cM based on the maize reference map 'Genetic 2008' (available at http://www.maizegdb.org/map.php). Coverage of the IL was computed by summing unique Gaspé introgressions across the IL. Introgression start- and end-points were taken as the mid-points from/to the nearest evaluated markers or the telomeres. The same approach was used to estimate the proportion of the Gaspé genome polymorphic with B73 and not represented in the library. Chromosome regions containing four or more adjacent monomorphic SSR markers were also similarly sized and considered technically non-representable (see Results).

### Production of the F_2 _and BC_1 _populations and linkage maps

Along with the IL, an F_2 _population and a BC_1 _population were produced and utilized for linkage map construction and QTL analysis. The 88 (B73 × Gaspé Flint) × B73 BC_1 _plants corresponded to the ones used to establish the IL and were genotyped with 70 SSRs evenly spread on the 10 maize chromosome. Linkage map construction was carried out using JoinMap 3.0 [64], with a LOD threshold for linkage grouping of 3.0 and produced 10 linkage groups which covered 1278 cM (71% of the reference map). The B73 × Gaspé Flint F_2 _population included 65 plants and was genotyped with 26 polymorphic SSR markers covering maize chromosomes 1, 3, 8, 9 and 10. This linkage map covered 641 cM, corresponding to 78% of the same five linkage groups from the reference maize map 'Genetic 2008'. For the two maps, marker order was always in agreement with the one in the reference map.

### Field experiment and collection of phenotypic data

All field experiments were carried out at the experimental station of the University of Bologna, near Cadriano (Lat.: N 44°33'03'', Lon.: E 11°24'36'', 33 m altitude), Italy. For the IL, 77 genotypes (75 IL lines, B73 and B73 × Gaspè Flint F_1_) were planted following a randomized complete block design, with four repetitions. Gaspé Flint was grown in adjacent plots and not included in the IL experiment because its reduced stature and vigor would have biased the phenotypic score of neighbouring plots. The experiment was planted on April, 20, 2006, in 1.4 m-long and 0.8 m-wide single-row plots, with eight plants per row, for a final density of 6.25 plants m^-2^. Nine phenotypic traits were recorded or computed (Table 1): number of days from planting to pollen shed (DPS) measured when 50% of the plants in a plot had extruded at least one anther; number of ears (EARN) per plant counted at flowering; growing degree units (GDU) as alternative score for flowering time as described (Equation 1 in [65]); number of plant nodes (ND), measured as total number of leaves at flowering (the 6^th ^and 12^th ^leaves were marked to account for the early developed leaves not visible at flowering); number of nodes below the top ear (NDBE) were counted with reference to the 6^th ^and/or the 12^th ^marks, including the leaf (node) of the top ear; number of nodes above the top ear (NDAE) were computed as ND - NDBE; proportion of nodes below the ear (PNDBE) was computed as NDBE/ND; plant height (PH) measured at the tip of the tassel; internode length (INDL) computed as the ratio PH/ND. The F_2 _population was grown contemporary and adjacent to the IL experiment, in plots of equal size. Phenotypic traits recorded or computed were the same as for the IL experiment. The BC_1 _population was planted on May 28, 2002, in 3 m-long and 1 m-wide plots, 0.2 m between plants (corresponding to 5 plants m^-2^). Phenotypic traits recorded or computed were DPS, EARN, GDU, INDL, ND and PH, as described for the IL experiment, while NDBE, NDAE and PNDBE were not considered.

### Phenotypic data and QTL analyses

Normality of distribution for all trait values was tested by the Shapiro-Wilk test implemented in GenStat11 (VSN international Ltd, UK). For the IL data, ANOVA (Kruskall-Wallis for not normally distributed traits), phenotypic and genotypic correlations and heritability were computed using PLABSTAT version 3A [66]. IL line mean values for normally distributed traits (DPS, EARN, GDU, INDL, PH and PNDBE) were compared with the B73 mean values following a two-sided Dunnet's test as implemented in Statistica 8.0 (StatSoft, Inc.), with an alpha level of 0.05, while for non-normally distributed traits (ND, NDBE and NDAE) a Steel's test was carried out as implemented in GenStat11. We additionally tested for the presence of a QTL in a given chromosome interval by means of a "bin mapping" approach where IL lines sharing the same introgression were grouped and the trait mean value tested against B73. Because in several cases the same introgression was shared by several lines, such test for the presence of a QTL was expected to have more statistical power than the simple comparison of each single line with B73. For normally distributed traits, bin mapping was carried out by a two-sided *t*-test, assuming unequal variance, as described in [9]. For the non-normally distributed traits, the non-parametric Mann-Whitney U-test was adopted instead. Both the *t*- and the U-tests were carried out using GenStat11.

A QTL for a given trait was declared to correspond to a Gaspé Flint chromosome introgression (marked by SSR markers), when i) more than one IL line carrying such introgression significantly differed from B73 and no other Gaspè Flint introgression was shared by the same IL lines, or ii) a single IL line was significantly different from B73 and QTL evidence was obtained at the same chromosome region by the BC_1 _or the F_2 _QTL analyses with concordance of the genetic effect direction. In this case, correspondence was declared when the introgressed chromosome region and the 2-LOD support interval overlapped and the F_2 _and/or the BC_1 _QTL LOD peak was included in the IL introgression region. The genetic effect for each QTL was computed as (m_ILL _- m_B73_)/2, were m_ILL _was the mean value for the IL line(s) with a given introgression and m_B73 _was the B73 value.

For the BC_1 _and the F_2 _datasets, QTL analyses was carried out using Composite Interval Mapping as implemented in PLABQTL [67]. The default cofactor selection parameters (cov SELECT option with a log of the likelihood (LOD) score of > 3.0 to enter into the model) were used. The maps were scanned at 1-centimorgan (cM) intervals. LOD threshold for QTL significance (*P *< 0.05) were obtained after 1,000 permutations and ranged from 2.94 for PH in the F_2 _population to 3.20 for PNDBE in the BC_1 _population. The reported genetic effects and the proportion of phenotypic variance explained (PVE) by each QTL were obtained after fitting a multi-QTL model with the 'final simultaneous fitting' routine. QTL positions (corresponding to relevant introgression regions identified in the IL experiment and to the 2-LOD drop supporting intervals computed for the F_2 _and BC_1 _populations) were projected using MapChart 2.2 [68] with cM distances based on the maize reference map 'Genetic 2008' available at http://www.maizegdb.org. QTL nomenclature followed rules proposed by [69].

## Authors' contributions

SS conceived the study, participated to the field and molecular work and drafted the manuscript; CSimona, BM, CN, CSara performed field and molecular work and participated to statistical analyses; SMC supervised field experiment design and performed statistical analysis; TR participated in conceiving the study, coordinating the field and molecular work and in drafting the manuscript. All authors read and approved the final manuscript.

## Supplementary Material

Additional file 1 **Frequency distributions of the IL, BC_1 _and F_2 _populations for the analysed traits**. This figure summarizes the frequency distribution for days to pollen shed (DPS), number of ears (EARN), growing degree units (GDU), internode length (INDL), number of nodes (ND), number of nodes below the top ear (NDBE), number of nodes above the ear (NDAE), plant height (PH) and proportion of nodes below the ear (PNDBE), for the three populations under study. Click here for file

Additional file 2 **Table reporting the phenotypic correlations among traits based on the B73 × Gaspé Flint BC_1 _population**. Click here for file

Additional file 3 **Table reporting the phenotypic correlation among traits based on the B73 × Gaspé Flint F_2 _population**. Click here for file

Additional file 4 **Table reporting the QTLs identified in the B73 × Gaspé Flint BC_1 _population**. Click here for file

Additional file 5 **Table reporting the QTLs identified in the B73 × Gaspé Flint F_2 _population**. Click here for file

Additional file 6 **Table reporting the SSR markers (and relative positions) screened for polymorphism between B73 and Gaspé Flint**. Click here for file
