# Supplementary material for: Genetic dissection of maize phenology using an intraspecific introgression library
Source: BMC Plant Biol. 2011 Jan 6;11:4. doi: 10.1186/1471-2229-11-4 (PMC3025946; doi:10.1186/1471-2229-11-4)
Supplement: Additional file 1 — Frequency distributions of the IL, BC1 and F2 populations for the analysed traits. This figure summarizes the frequency distribution for days to pollen shed (DPS), number of ears (EARN), growing degree units (GDU), internode length (INDL), number of nodes (ND), number of nodes below the top ear (NDBE), number of nodes above the ear (NDAE), plant height (PH) and proportion of nodes below the ear (PNDBE), for the three populations under study. [file 1471-2229-11-4-S1.DOC]

**Additional file 1 - Frequency distributions of the IL, BC_1_ and F_2_ populations for analysed traits**

Histograms summarizing the frequency distribution for days to pollen shed (DPS), number of ears (EARN), growing degree units (GDU), internode length (INDL), number of nodes (ND), number of nodes below the top ear (NDBE), number of nodes above the ear (NDAE), plant height (PH) and proportion of nodes below the ear (PNDBE), for the three populations under study. The letters B, F_1_ and G (and linked black arrows) indicate the phenotypic mean values for B73, B73 × Gaspé Flint F_1_ and Gaspé Flint, respectively. The parental values for the F_2_ and IL histograms refer to the same values as the two populations were integrated in the same field experiment.
