# Supplementary material for: Genetic dissection of maize phenology using an intraspecific introgression library
Source: BMC Plant Biol. 2011 Jan 6;11:4. doi: 10.1186/1471-2229-11-4 (PMC3025946; doi:10.1186/1471-2229-11-4)
Supplement: Additional file 2 — Table reporting the phenotypic correlations among traits based on the B73 × Gaspé Flint BC1 population. [file 1471-2229-11-4-S2.DOC]

**Additional file 2** - Phenotypic correlations among traits based on the BC_1_ population.

|  | DPS | EARN | GDU | INDL | ND |
| --- | --- | --- | --- | --- | --- |
| EARN | 0.38** | - |  |  |  |
| GDU | 0.99** | 0.39** | - |  |  |
| INDL | -0.45** | -0.25* | -0.45** | - |  |
| ND | 0.76** | 0.41** | 0.77** | -0.59** | - |
| PH | 0.65** | 0.33** | 0.65** | -0.08 | 0.84** |

* and ** Significant at *P* 0.05 and 0.01, respectively.
