# Supplementary material for: Genetic dissection of maize phenology using an intraspecific introgression library
Source: BMC Plant Biol. 2011 Jan 6;11:4. doi: 10.1186/1471-2229-11-4 (PMC3025946; doi:10.1186/1471-2229-11-4)
Supplement: Additional file 3 — Table reporting the phenotypic correlation among traits based on the B73 × Gaspé Flint F2 population. [file 1471-2229-11-4-S3.DOC]

**Additional file 3 -** Phenotypic correlations among traits based on the F_2_ population

|  | DPS | EARN | GDU | INDL | ND | NDAE | NDBE | PH |
| --- | --- | --- | --- | --- | --- | --- | --- | --- |
| EARN | 0.18 | - |  |  |  |  |  |  |
| GDU | 0.99** | 0.18 | - |  |  |  |  |  |
| INDL | -0.36** | -0.18 | -0.39** | - |  |  |  |  |
| ND | 0.83** | 0.15 | 0.85** | -0.52** | - |  |  |  |
| NDAE | 0.58** | 0.08 | 0.58** | -0.53** | 0.76** | - |  |  |
| NDBE | 0.85** | 0.26 | 0.86** | -0.58** | 0.93** | 0.47** | - |  |
| PH | 0.69** | 0.05 | 0.68** | 0.10 | 0.79** | 0.57** | 0.73** | - |
| PNDBE | 0.15 | 0.14 | 0.16 | 0.05 | 0.04 | -0.61** | 0.41** | 0.08 |

** Significant at *P* 0.01.
