# Supplementary material for: Genetic dissection of maize phenology using an intraspecific introgression library
Source: BMC Plant Biol. 2011 Jan 6;11:4. doi: 10.1186/1471-2229-11-4 (PMC3025946; doi:10.1186/1471-2229-11-4)
Supplement: Additional file 4 — Table reporting the QTLs identified in the B73 × Gaspé Flint BC1 population. [file 1471-2229-11-4-S4.DOC]

**Additional file 4** - Parameters of the QTLs identified in the B73 × Gaspé Flint BC_1_ population

| Trait | Bin | cM ^a^ | Marker | Effect ^b^ | LOD | PVE ^c^ |
| --- | --- | --- | --- | --- | --- | --- |
| DPS | 1.03-04 | 55-85-114 | phi001 | -2.8 | 3.0 | 13.2 |
|  | 3.04-05 | 58-72-98 | umc1223 | -4.0 | 3.0 | 31.1 |
|  | 8.05 | 75-92-99 | umc1846 | -4.8 | 11.4 | 40.0 |
| GDU | 1.03-04 | 57-85-114 | phi001 | -35.7 | 3.6 | 13.8 |
|  | 3.04-05 | 58-67-97 | umc1223 | -49.6 | 3.7 | 30.3 |
|  | 8.05 | 75-92-99 | umc1846 | -60.3 | 11.3 | 40.2 |
| INDL | 5.06-08 | 115-141-159 | bnlg609 | 2.2 | 3.2 | 15.0 |
|  | 8.05-07 | 90-105-114 | umc1149 | 1.3 | 2.6 | 12.3 |
| NDBE | Trait not analyzed | | | | | |
| NDAE | Trait not analyzed | | | | | |
| ND | 3.04-06 | 58-85-97 | umc1167 | -2.2 | 5.3 | 34.3 |
|  | 4.03-05 | 39-61-69 | bnlg490 | -1.0 | 2.5 | 10.4 |
|  | 8.05 | 71-83-97 | bnlg1863 | -2.9 | 16.3 | 52.0 |
| PH | 3.03-06 | 30-50-77 | bnlg1647 | -30.3 | 4.5 | 32.7 |
|  | 4.03-04 | 36-58-64 | umc1509 | -16.4 | 3.1 | 14.5 |
|  | 8.05 | 63-79-87 | bnlg1863 | -39.3 | 16.7 | 49.2 |
|  | 10.01-04 | 10-44-63 | bnlg1451 | 6.5 | 3.2 | 1.5 |

^a^ The three digits indicate the left limit of the QTL supporting interval (SI), the LOD peak position and the right limit of QTL SI, respectively.

^b^ Computed as H – B, where H is ‘heterozygous Gaspé Flint/B73’ and B is ‘homozygous B73’.

^c^ Proportion of phenotypic variance explained by the QTL after fitting a multiple QTL model.
