# Supplementary material for: Genetic dissection of maize phenology using an intraspecific introgression library
Source: BMC Plant Biol. 2011 Jan 6;11:4. doi: 10.1186/1471-2229-11-4 (PMC3025946; doi:10.1186/1471-2229-11-4)
Supplement: Additional file 5 — Table reporting the QTLs identified in the B73 × Gaspé Flint F2 population. [file 1471-2229-11-4-S5.DOC]

**Additional file 5** - Parameters of the QTLs identified in the B73 × Gaspé Flint F_2_ population.

| Trait | Bin | cM ^a^ | Marker | Effect ^b^ | LOD | PVE ^c^ |
| --- | --- | --- | --- | --- | --- | --- |
| DPS | 1.02 | 26-26-50 | umc1685 | -1.8 | 2.9 | 15.4 |
|  | 8.05 | 78-90-97 | umc1846 | -4.7 | 12.0 | 46.3 |
|  | 10.04 | 65-81-85 | umc2163 | -3.2 | 4.6 | 29.3 |
| GDU | 1.02 | 26-26-50 | umc1685 | -24.0 | 3.3 | 17.4 |
|  | 8.05 | 80-90-97 | umc1846 | -57.0 | 12.0 | 44.8 |
|  | 10.04 | 70-81-85 | umc2163 | -42.1 | 4.9 | 31.1 |
| INDL | 9.02-04 | 29-66-83 | umc1271 | 2.1 | 5.0 | 18.8 |
| ND | 8.05 | 78-97-105 | umc1846 | -1.5 | 7.9 | 27.1 |
|  | 9.02-04 | 25-63-86 | umc1271 | -1.0 | 3.1 | 15.0 |
|  | 10.04 | 70-81-85 | umc2163 | -1.8 | 4.7 | 36.3 |
| NDBE | 8.05 | 76-93-100 | umc1846 | -1.1 | 7.0 | 29.1 |
|  | 9.02-04 | 36-77-86 | umc1271 | -0.9 | 4.1 | 11.3 |
|  | 10.04-06 | 70-85-105 | bnlg1250 | -1.2 | 3.4 | 34.8 |
| NDAE | 10.04-06 | 66-81-111 | umc2163 | -0.7 | 2.8 | 20.7 |
| PH | 3.04-05 | 50-76-106 | bnlg1647 | -28.1 | 5.8 | 29.7 |
|  | 8.05 | 67-90-104 | bnlg1863 | -22.1 | 3.8 | 24.5 |
|  | 8.06-08 | 115-143-143 | umc1149 | -18.4 | 3.3 | 20.3 |
|  | 10.05-06 | 85-105-111 | bnlg1250 | -27.9 | 3.7 | 33.0 |

^a^ The three digits indicate the left limit of the QTL supporting interval (SI), the LOD peak position and the right limit of QTL SI, respectively.

^b^ Computed as (Gaspé Flint–B73)/2.

^c^ Proportion of phenotypic variance explained by the QTL after fitting a multiple QTL model.
