# Supplementary material for: Genetic dissection of maize phenology using an intraspecific introgression library
Source: BMC Plant Biol. 2011 Jan 6;11:4. doi: 10.1186/1471-2229-11-4 (PMC3025946; doi:10.1186/1471-2229-11-4)
Supplement: Additional file 6 — Table reporting the SSR markers (and relative positions) screened for polymorphism between B73 and Gaspé Flint. [file 1471-2229-11-4-S6.DOC]

**Additional file 6**. List and mapping information of SSR markers screened for polymorphism between B73 and Gaspé Flint.

| Marker | Polimorphism  B73 *vs*. Gaspé Flint | Bin | Position ‘Genetic 2008’ ^1^ |
| --- | --- | --- | --- |
| umc1948 | yes | 1.01 | 22.9 |
| umc1685 | yes | 1.01 | 25.8 |
| umc1976 | no | 1.02 | 40.2 |
| bnlg1953 | no | 1.02 | 42.5 |
| bnlg1007 | yes | 1.02 | 43.3 |
| phi001 (ts2) | yes | 1.03 | 63.9 |
| bnlg2295 | unclear | 1.04 | 99.6 |
| umc1903 | yes | 1.05 | 117 |
| umc1395 | yes | 1.05 | 117.9 |
| umc1122 | yes | 1.06 | 148.4 |
| umc1358 | yes | 1.07 | 163.4 |
| umc1013 (umc128) | yes | 1.08 | 180.6 |
| dupssr12 | no | 1.08 | - |
| mmc0041 | no | 1.08 | 196.9 |
| bnlg1643 | yes | 1.08 | 200.7 |
| umc2047 | yes | 1.09 | 210.6 |
| umc1306 | yes | 1.09 | 220.3 |
| bnlg1671 | yes | 1.10 | 228.4 |
| umc1064 (fdx3) | yes | 1.11 | 274.6 |
| umc2186 | no | 2.00 | - |
| umc1622 | yes | 2.00 | 0.7 |
| umc2094 | yes | 2.01 | - |
| umc1165 | yes | 2.01 | 11.9 |
| umc1552 | no | 2.01-2.02 | 14.9 |
| umc1823 | yes | 2.02 | 23.2 |
| umc1261 | no | 2.02 | 38.7 |
| mmc0231 | yes | 2.03 | 44.9 |
| bnlg1064 | yes | 2.03 | 56.8 |
| bnlg381 | yes | 2.04 | 61.2 |
| bnlg1063 | no | 2.05 | 77.4 |
| umc2030 | yes | 2.04 | 78.4 |
| umc1454 | unclear | 2.04 | 84.8 |
| umc1635 | yes | 2.04 | 86.2 |
| umc1884 | yes | 2.05 | 87 |
| bnlg1047 | no | 2.05 | 96.2 |
| nc003 | no | 2.06 | - |
| umc1875 | yes | 2.06 | 94.8 |
| umc1080 | no | 2.06 | 95 |
| umc1004 | no | 2.06 | 95.5 |
| umc1108 | unclear | 2.06 | 100.4 |
| mmc0271 | no | 2.07 | 109.6 |
| bnlg1045 | yes | 2.07 | 117.8 |
| umc1798 | yes | 2.08 | 137.8 |
| bnlg1520 | yes | 2.09 | 151.5 |
| umc2071 | yes | 3.01 | 7.3 |
| umc2257 | yes | 3.01 | 7.6 |
| umc2049 | yes | 3.01 | 9.7 |
| umc1458 | yes | 3.02 | 16.8 |
| bnlg1647 | yes | 3.02 | 25.8 |
| bnlg1447 | yes | 3.03 | 32.4 |
| umc1030 | yes | 3.04 | 39.8 |
| umc1900 | no | 3.04 | 58.1 |
| umc1223 | yes | 3.04 | 58.6 |
| umc2155 | no | 3.05 | - |
| umc1167 | yes | 3.05 | 79.8 |
| umc1266 | yes | 3.06 | 102.9 |
| bnlg1350 | yes | 3.08 | 111 |
| umc2271 | yes | 3.06 | 123.5 |
| umc1528 | yes | 3.07 | 136.1 |
| mmc0251 | no | 3.08 | 152.8 |
| umc2008 | no | 3.09 | 186.8 |
| bnlg1754 | no | 3.09 | 189.3 |
| umc1594 | no | 3.09 | 207.2 |
| umc2048 | no | 3.10 | 214.7 |
| umc2148 | unclear | 4.01 | 5.9 |
| umc1682 (cyp5) | yes | 4.01 | 11.9 |
| umc1509 | yes | 4.02 | 25 |
| umc1943 | no | 4.02 | 25.3 |
| umc1288 | no | 4.02 | 26.8 |
| umc1294 | no | 4.02 | 27.1 |
| umc2281 | no | 4.03 | 39.7 |
| umc2176 | no | 4.03 | 43.7 |
| umc2039 | no | 4.03 | 49.1 |
| umc1821 | no | 4.04 | 53.6 |
| umc2206 | no | 4.04 | 58.7 |
| bnlg490 | yes | 4.04 | 59.5 |
| phi026 (gpc1) | no | 4.05 | 63.5 |
| bnlg1265 | yes | 4.05 | 67.1 |
| bnlg2291 | yes | 4.06 | 98.1 |
| umc1808 | yes | 4.08 | 113.2 |
| umc1940 | yes | 4.09 | 145.5 |
| umc2011 | no | 4.10 | 172.3 |
| bnlg1917 | unclear | 4.10 | 175.9 |
| umc1491 | no | 5.00 | -8.1 |
| umc2292 | no | 5.00 | 5.8 |
| bnlg1006 | no | 5.00 | 11.9 |
| umc1523 | no | 5.01 | 18 |
| bnlg1836 | no | 5.01 | 26.5 |
| umc1781 | no | 5.01 | 31.2 |
| umc1587 | yes | 5.02 | 39.2 |
| bnlg105 | no | 5.02 | 46.3 |
| umc2293 | yes | 5.03 | 49.2 |
| umc1852 | no | 5.03 | 53.5 |
| umc1705 | yes | 5.03 | 60.2 |
| umc1221 | yes | 5.04 | 92.1 |
| mmc0081 | yes | 5.05 | 97.5 |
| umc1019 (umc126a) | yes | 5.06 | 117.4 |
| bnlg609 | yes | 5.06 | 125.2 |
| bnlg1346 | no | 5.07 | 136.2 |
| bnlg2305 | no | 5.07 | 139.3 |
| bnlg1306 | no | 5.07 | 139.3 |
| umc1225 | yes | 5.08 | 160.4 |
| bnlg1371 | no | 6.01 | 18.2 |
| umc1625 | no | 6.01 | 20.4 |
| umc1006 | no | 6.02 | 31.3 |
| phi077 | no | 6.01 | 32.3 |
| bnlg2191 | yes | 6.02 | 37.2 |
| umc1887 | no | 6.03-6.04 | 41.7 |
| umc1014 | yes | 6.04 | 52.9 |
| umc2006 | yes | 6.04 | 57.2 |
| umc1751 | no | 6.05 | 63.6 |
| umc1413 | yes | 6.05 | 69.3 |
| bnlg1702 | yes | 6.05 | 80.2 |
| umc1859 | yes | 6.06 | 97.9 |
| umc1653 | yes | 6.07 | 133.7 |
| umc1241 | yes | 7.00 | 3.5 |
| bnlg1337 | yes | 7.00 | 3.8 |
| umc2160 (mmp81) | yes | 7.01 | 29 |
| bnlg2160 | no | 7.01 | 33.2 |
| umc1036 | yes | 7.02 | 37.8 |
| umc2236 | yes | 7.02 | 38.8 |
| umc1927 | yes | 7.02 | 42 |
| bnlg1792 | yes | 7.02 | 47.6 |
| umc1983 | yes | 7.02 | 61.1 |
| bnlg1022 | yes | 7.02 | 69.5 |
| umc1567 | yes | 7.03 | 75 |
| umc1015 (php20569a) | no | 7.03 | 75 |
| umc1456 | unclear | 7.03 | 82.4 |
| umc1660 | no | 7.03 | 87.9 |
| umc1841 | yes | 7.03 | 95.2 |
| umc1001 | yes | 7.03 | 101.8 |
| umc1029 | yes | 7.04 | 111.2 |
| umc1342 | no | 7.04 | 111.2 |
| umc1593 | yes | 7.04 | 112.6 |
| umc1154 | yes | 7.05 | 139.7 |
| umc1406 | no | 7.05 | 149.7 |
| umc1760 | no | 7.05 | 151.9 |
| umc1786 | no | 8.01 | 2.7 |
| umc1592 | yes | 8.01 | 8.5 |
| umc1817 | yes | 8.02 | 28.8 |
| umc1807 | yes | 8.03 | 51.5 |
| umc1984 | no | 8.03 | 58.7 |
| bnlg1863 | yes | 8.03 | 61.4 |
| mite | yes | 8.05 | - |
| umc1846 | yes | 8.05 | 89.5 |
| bnlg1651 | yes | 8.05 | 93.6 |
| umc1149 | yes | 8.06 | 103.5 |
| umc1724 | yes | 8.07 | 114.3 |
| umc2014 | no | 8.07 | 115.8 |
| umc1384 | no | 8.07 | 126.9 |
| umc1005 | no | 8.08 | 131.7 |
| umc1032 | no | 8.08 | 134.7 |
| phi015 (gst1) | yes | 8.08 | 142.9 |
| umc1040 | yes | 9.01 | 5.3 |
| bnlg1583 | no | 9.01 | 15.6 |
| umc1596 | no | 9.01 | 21.7 |
| umc1170 | yes | 9.02 | 25.3 |
| umc2219 | no | 9.02 | 31.9 |
| bnlg244 | no | 9.02 | 35.7 |
| bnlg1401 | no | 9.02 | 36.9 |
| umc1037 | no | 9.02 | 40 |
| umc1586 | no | 9.03 | 49.9 |
| umc1191 | no | 9.03 | 58.2 |
| umc1271 | yes | 9.03 | 60.1 |
| umc1921 | no | 9.03 | 62.4 |
| umc1771 | yes | 9.04 | 79.3 |
| umc1519 | no | 9.04 | 80.2 |
| umc1231 | yes | 9.05 | 85.5 |
| bnlg1525 | yes | 9.07 | 134.2 |
| umc1293 | yes | 10.00 | 11.1 |
| bnlg1451 | yes | 10.02 | 18.6 |
| umc2053 | no | 10.01 | 19.1 |
| bnlg1712 | no | 10.03 | 54.5 |
| bnlg1655 | no | 10.03 | 57 |
| umc2180 | no | 10.03-04 | 57.1 |
| umc2163 (mmp121) | yes | 10.04 | 67.4 |
| bnlg1250 | yes | 10.05 | 83.9 |
| bnlg1185 | no | 10.05 | - |
| umc2043 | no | 10.05 | - |
| umc1084 | yes | 10.07 | 111.4 |

^1^ Based on the maize reference map ‘Genetic 2008’ available at www.maizegdb.org/map.php
